# Barriers and facilitators of vaccine hesitancy for COVID-19, influenza, and pertussis during pregnancy and in mothers of infants under two years: An umbrella review

**DOI:** 10.1371/journal.pone.0282525

**Published:** 2023-03-02

**Authors:** Bethany Nichol, Jemma Louise McCready, Mary Steen, John Unsworth, Valentina Simonetti, Marco Tomietto

**Affiliations:** 1 Department of Social Work, Education and Community Wellbeing, Faculty of Health and Life Sciences, Northumbria University, Newcastle upon Tyne, United Kingdom; 2 Department of Nursing, Midwifery and Health, Faculty of Health and Life Sciences, Northumbria University, Newcastle upon Tyne, United Kingdom; 3 Department of Biomedical Science and Human Oncology, University of Bari "Aldo Moro", Bari, Italy; 4 Research Unit of Nursing Science and Health Management, University of Oulu, Oulu, Finland; 5 Visiting Professor, University of Bari “Aldo Moro”, Bari, Italy; Tehran University of Medical Sciences, ISLAMIC REPUBLIC OF IRAN

## Abstract

**Background:**

Vaccination during pregnancy has been repeatedly demonstrated to be safe and effective in protecting against infection and associated harms for the mother, developing baby, and subsequent infant. However, maternal vaccination uptake remains low compared to the general population.

**Objectives:**

An umbrella review to explore the barriers and facilitators to Influenza, Pertussis and COVID-19 vaccination during pregnancy and within 2 years after childbirth, and to inform interventions to encourage uptake (PROSPERO registration number: CRD42022327624).

**Methods:**

Ten databases were searched for systematic reviews published between 2009 and April 2022 exploring the predictors of vaccination or effectiveness of interventions to improve vaccination for Pertussis, Influenza, or COVD-19. Both pregnant women and mothers of infants under two years were included. Barriers and facilitators were organised using the WHO model of determinants of vaccine hesitancy through narrative synthesis, the Joanna Briggs Institute checklist assessed review quality, and the degree of overlap of primary studies was calculated.

**Results:**

19 reviews were included. Considerable overlap was found especially for intervention reviews, and the quality of the included reviews and their primary studies varied. Sociodemographic factors were specifically researched in the context of COVID-19, exerting a small but consistent effect on vaccination. Concerns around the safety of vaccination particularly for the developing baby were a main barrier. While key facilitators included recommendation from a healthcare professional, previous vaccination, knowledge around vaccination, and communication with and support from social groups. Intervention reviews indicated multi-component interventions involving human interaction to be most effective.

**Conclusion:**

The main barriers and facilitators for Influenza, Pertussis and COVID-19 vaccination have been identified and constitute the foundation for policy development at the international level. Ethnicity, socioeconomic status, concerns about vaccine safety and side effects, and lack of healthcare professionals’ recommendations, are the most relevant factors of vaccine hesitancy. Adapting educational interventions to specific populations, person-to-person interaction, healthcare professionals’ involvement, and interpersonal support are important strategies to improve uptake.

## Introduction

Vaccine hesitancy is defined as the delay in accepting or rejecting safe vaccines despite the availability of vaccination services and it describes a continuum between undecided and anti-vaccination behaviors [1]. Vaccine hesitancy is higher in women (21%) than in men (14.7%) [1]. Vaccination coverage is particularly low among pregnant women, with average uptake rarely exceeding 50% for Pertussis [2], Influenza [2, 3] and COVID-19 [4]. Uptake is especially low in women of lower socioeconomic status [5]. This is a public health concern as pregnant women are a vulnerable population and, if unvaccinated, are at higher risk of hospitalisation [6] or even death during pregnancy [7], with an increased chance of pre-eclampsia [8], the need for emergency caesarean section [6, 8], and stillbirth [6] from contracting COVID-19. Pertussis and Influenza pose a threat to the unborn child, as mortality is highest in infants under six months [9, 10]. Maternal vaccination provides a safe [11–16] and cost effective [13, 17] way to protect the mother and infant from contracting both Influenza [17] and Pertussis [13], reducing preterm births [14, 18], low birth weight [18], and stillbirths [15]. Although the evidence on COVID-19 vaccination during pregnancy is in its infancy, emerging evidence supports the safety of vaccination [19].

The World Health Organisation (WHO) SAGE group ‘model of determinants for vaccine hesitancy’ [20] classifies vaccination behaviour into three domains [21]: contextual influences (relating to one’s wider sociodemographic, political, institutional, or economic background)(I), individual and group influences (including social environment, attitudes, and beliefs)(II), and vaccine and vaccine-specific issues (relating to access or the vaccination process)(III). Maternal vaccination for COVID-19, Influenza, and Pertussis are currently recommended by key advisory bodies such as the Advisory Committee on Immunization Practices in the United States [19, 22, 23]. Additionally, the COVID-19 Vaccine Global Access Federation Facility (COVAX) aims to ensure equitable access to affordable vaccination across the world [24] in partnership the World Health Organisation (WHO). Following increased availability and access, individual level determinants of vaccine hesitancy have become most important for investigating differences in uptake of maternal vaccination.

The determinants of COVID-19 vaccination hesitancy differ in comparison to other maternal vaccinations. For example, the influence of mass media and fake news have led to concerns about vaccine safety for the mother and the baby, and a mistrust to healthcare professionals’(HCPs) recommendations or institutional guidelines [25]. These factors are amplified by certain demographic characteristics such as identifying as an ethnic minority [26]. Understanding the determinants of vaccine hesitancy during pregnancy specifically and how these differ between vaccinations is a pre-requisite to developing interventions to increase uptake. Further, comparison of the findings from COVID-19 and Influenza after the Influenza pandemic in 2009 is beneficial in forecasting not only future COVID-19 action but reactions to future pandemics too.

A synthesis of the evidence exploring vaccine hesitancy during pregnancy over the past decade is required. Given the numerous primary studies and reviews available on this topic, an umbrella review offers a broad scope to provide an overall picture of the evidence, whilst assessing its quality and credibility [27]. The aim of this umbrella review is therefore to synthesise the established literature on Influenza with the emerging literature on COVID-19, to assess the most influential barriers and facilitators to vaccination during pregnancy.

## Methods

The protocol for this umbrella review was registered via PROSPERO [28], (registration number: CRD42022327624). The PRISMA guidelines for reporting were used throughout [29].

### Inclusion exclusion criteria

Systematic reviews of quantitative or mostly quantitative studies with or without meta-analysis were included that investigated the barriers and facilitators to vaccine hesitancy, either for COVID-19, Influenza, Pertussis, or a combination. Study samples were required to be pregnant or have been pregnant within the past two years, and either not vaccinated or partially unvaccinated.

### Search strategy

A systematic search was performed on 22^nd^ April 2022 (and last updated on the 3^rd^ January 2023) on ten databases including EPISTEMONIKOS, CINAHL via EPSCO, and PsychArticles and The Health Research Collection via ProQuest (Consumer Health Database, Health & Medical Collection, Healthcare Administration Database, MEDLINE®, Nursing & Allied Health, Database, Psychology Database, and Public Health Database). Published systematic reviews were peer-reviewed, published in English, and from 2009 onwards. This cut-off was reflective of the Influenza pandemic in 2009 [30]. 2009 is also significant as the first RCT demonstrating the effectiveness of the seasonal Influenza vaccine during pregnancy had been published [31]. A library information specialist was consulted to check the search strategy against the PRESS statement [32]. The search strategy (Table 1, see also Supplementary Material 1 in S1 File for specific search queries) was created in accordance with the inclusion criteria and combined with Boolean operators ‘AND’ and ‘OR’. Search terms were divided into the topic of vaccination, the type of vaccination, specifying vaccine hesitancy, and the population of pregnant women. Discussion with other academics and forward and backward citation searching was applied to ensure a comprehensive search. As the Pertussis vaccine is often combined together with Tetanus and Diphtheria vaccine (Tetanus, Diphtheria And Pertussis–TDAP vaccine) and recommended during pregnancy, the TDAP vaccine has been specifically included in the search strategy. However, the main target of this vaccine during pregnancy is Pertussis, and, in some countries, it is not combined with Tetanus and Diphtheria. For this reason, this umbrella review specifically focused on Pertussis.

**Table 1:** A summary of the search strategy, each column was divided by the Boolean operator ‘AND’ during the search.

Vaccination	Hesitancy	For: COVID, Influenza and Pertussis	In pregnant women
vaccin* OR immunis* OR immuniz* OR incoculat*	Anxiet* OR doubt* OR trust* OR intent* OR dilemma* OR attitude* OR distrust OR mistrust OR controvers* OR objector* OR awareness OR dropout* OR Perception* OR misconception* OR uptake OR behavi*r OR exemption* OR refus* OR misinform* OR barrier* OR belief* OR fear* OR reject* OR oppos* OR choice* OR criticis* OR hesitanc* OR rumo*r OR delay OR accept* OR concern* OR knowledge OR confiden* OR decision OR anti-vaccin* OR predict* OR factors OR failure OR affect OR reason* OR utilis* OR utiliz* OR worry OR facilitate* OR enable* OR implement* OR frequency OR cause* OR willing* OR perspective* OR determine* OR react* OR indecision OR reluct*	Influenza OR H1N1 OR H5N1 OR flu OR TIV OR IIV3 OR IIV4 OR COVID OR COVID19 OR "SARS-CoV-2" OR "SARS-CoV2" OR SARSCoV2 OR "SARSCoV-2" OR "SARS coronavirus 2" OR "2019 nCoV" OR "2019nCoV" OR "2019-novel CoV" OR "nCov 2019" OR "nCov 19" OR "severe acute respiratory syndrome coronavirus 2" OR "novel coronavirus disease" OR "novel corona virus disease" OR "corona virus disease 2019" OR "coronavirus disease 2019" OR "novel coronavirus pneumonia" OR "novel corona virus pneumonia" OR "severe acute respiratory syndrome coronavirus 2" OR ‘whooping cough’ OR ‘Pertussis’ OR ‘tdap’	maternal OR antenatal OR prenatal OR pregnan* OR perinatal

### Study selection

Search results were downloaded from EPSCO and ProQuest via a RIS file and uploaded onto Rayyan [33] for screening. Following removal of duplicates, reviewer BN screened the remaining studies against the inclusion criteria based on title and abstract, followed by the remaining articles based on full text. For both of these stages, a second reviewer (JM) independently screened 10%, with any differences in decisions resolved through discussion.

### Quality appraisal

Quality appraisal for the included reviews were conducted by BN using The Joanna Brigg’s Institute (JBI) critical appraisal tool for systematic reviews and research syntheses [34], which examines reviews for trustworthiness and quality of findings using eleven distinct aspects. The eleven aspects were analyzed using “yes”, “no”, “unclear” and “not applicable” criteria. Rather than excluding reviews based on quality, quality was considered when synthesising results. Again, second reviewer JM independently conducted quality appraisal, with differences in conclusion resolved through discussion. Cohen’s kappa statistic [35] was calculated to judge the degree of agreement between reviewers, following the judgement parameters from Altman [36].

### Data extraction and synthesis

Data extraction followed guidance for overviews of reviews by Cochrane [37]. Data was extracted relating to study information, characteristics, search strategy, inclusion criteria, outcomes studied, findings, and conclusions. Reviewer JM completed data extraction for 10% of included studies, with any discrepancies resolved through discussion. Summarising rather than re-analysing the data was applied, in accordance to the nature of the research question [37]. Barriers and facilitators identified by the included reviews were mapped onto the WHO framework [38]. Heterogeneity statistics, pooled estimates, and 95% confidence intervals were collected where reviews included meta-analysis.

### Data analysis

Studies were first entered into a table mapping the primary studies contained within included systematic reviews, to analyse the degree of overlap between reviews. ‘Calculated covered area’ (CCA) was calculated to estimate degree of overlap in terms of a percentage, using the equation by Pieper et al. [39]. To aid in the discussion of the quality appraisal of included reviews by categorising reviews based on quality, the scoring system by Kilich et al. [40] was applied. Any item marked ‘yes’ scored two, items marked ‘no’ subtracted two, items marked ‘unclear’ subtracted one, and ‘not applicable’ did not affect the scores. Scores were categorised into very low, low, moderate, and strong. Overlap, quality of each review, and quality assessment of primary studies for the included reviews were mapped together onto one table.

## Results

### Search outcomes

**Fig 1:**
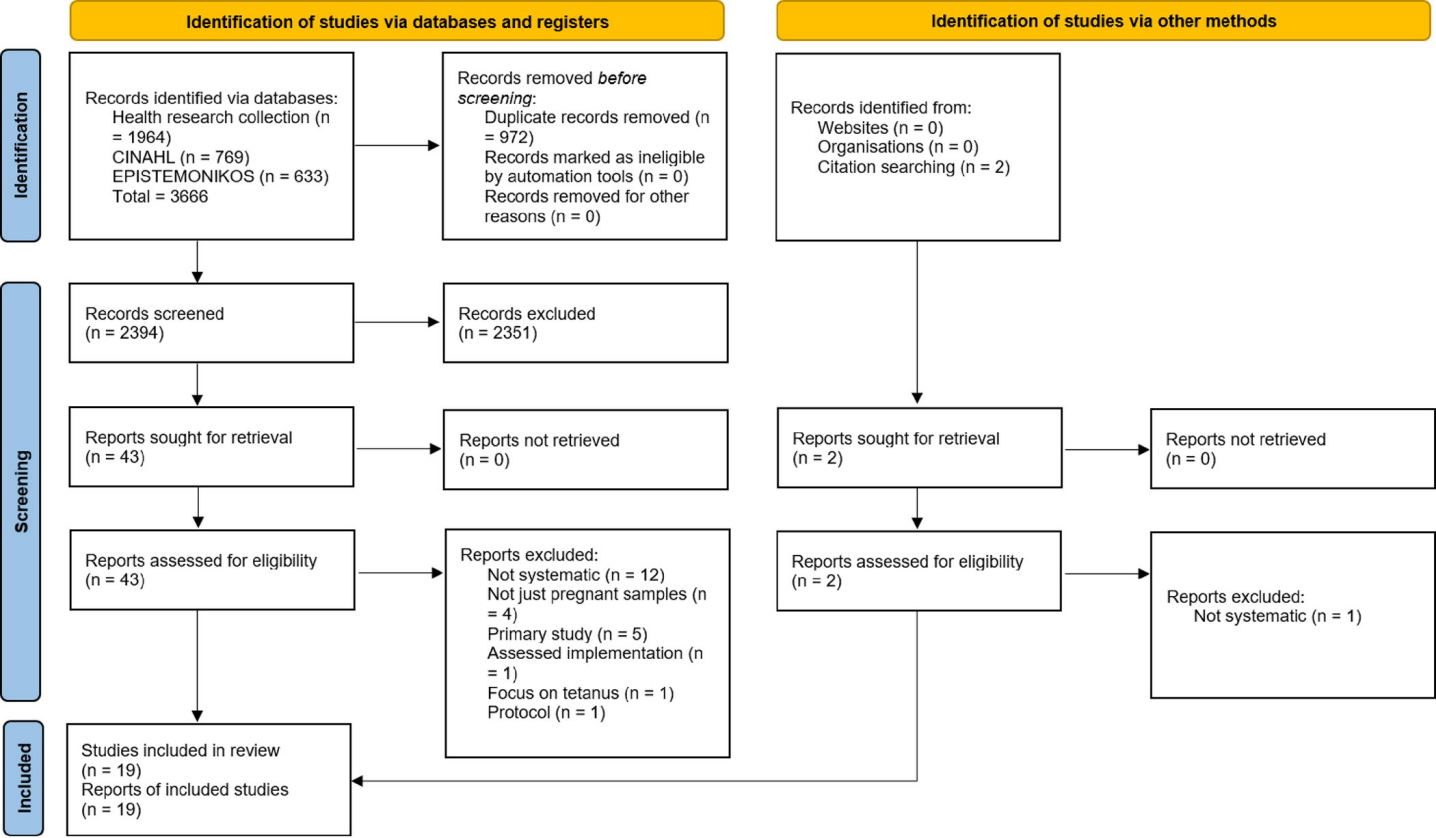
PRISMA 2020 diagram (Page et al., 2021), illustrating the exclusion process and distinguishing between article sources.

The PRISMA [29] diagram (Fig 1) illustrates the screening of 3366 retrieved articles. Articles were screened out based on full text for the following reasons; reviews were not systematic [41–52], samples were included other than pregnant samples [3, 53–55], the article was a primary study [56–60], the review described implementation rather than individual level barriers and facilitators [61], the focus was around tetanus vaccination [62], and the paper described a review protocol [63].

### Overlap

Degree of overlap in primary studies amongst included reviews was calculated separately for each of the following based on their exclusivity in the literature; barriers and facilitators related to COVID, barriers and facilitators related to all other included vaccinations, and reviews of interventions to increase vaccination. Including them in one overlap calculation would have artificially minimised the degree of overlap. Degree of overlap was found to be moderate for vaccination studies including Influenza, Pertussis and tetanus (7%), high in studies including COVID (15%), and very high for reviews assessing the effect of interventions (26%).

### Quality of included primary studies

13 reviews assessed included studies for either quality or risk of bias [40, 64–74], with the Newcastle-Ottawa scale and JBI quality assessments the most frequently used tools. Of the reviews that did assess for quality, their quality assessment is displayed alongside the review’s own quality appraisal and the assessment tool they used in Supplementary material 2 in S1 File. Interestingly, none of the primary studies of COVID-19 vaccination were judged to be as high risk of bias or of low quality. The quality and risk of bias assessments for Influenza and Pertussis were mixed and used a wide range of assessment tools.

### Quality appraisal of included reviews

The JBI score for included reviews ranged between -5 to 22 out of a possible 22. Included reviews were mostly rated at either extreme, but most rated strong quality. Seven were rated as very low [38, 69, 75–79], two low [64, 66], two as moderate [67, 70], and eight as strong [40, 65, 68, 71–74, 80]. As shown in Supplementary material 3 in S1 File, the main issues that led to low scoring was the lack of a protocol to clarify the research questions before screening, a minimal search strategy, missing details about data extraction, quality assessment, whether they were cross-checked, no validated quality assessment or test for publication bias, and a lack of direction for future research. Cohen’s kappa statistic was fair (*k* = .32, CI = -.20-.27, *p* = .013).

### Publication bias

The eight studies that assessed publication bias did so using funnel plots [40, 65, 67, 70, 72, 74], Egger’s [65, 67, 70, 73, 80] or Begg’s tests [70, 80]. Although several predictors did not acquire enough included studies to assess publication bias [40, 65, 74], reviews mostly concluded that there was no publication bias [65, 70, 73, 80].

### Review characteristics

The 19 included reviews (Table 2) can be divided into three main categories; nine assessed barriers and facilitators to COVID-19 [67, 69–71, 73, 77–80], four assessed barriers and facilitators to one or both of Influenza and Pertussis [38, 40, 64, 65], and six assessed the effectiveness of interventions to increase vaccination of one or both of Influenza and Pertussis [66, 68, 72, 74–76]. Of those in the latter two categories, six studied Influenza alone [64, 65, 72, 74–76], one studied Pertussis only [68], and three included both [38, 40, 66]. No reviews assessed the effects of interventions to increase COVID-19 vaccination, likely due to the short period from the start of the COVID-19 outbreak. Of the 16 reviews, eight included a meta-analysis [40, 65, 67, 69, 70, 72, 73, 80], of which five were concerning COVID-19 vaccination. Number of included studies ranged from three [80] to 155 [38], with reported sample sizes of included studies ranging from 10 [38] to 1, 862, 705 [65]. Although most reviews only included pregnant women, some included postpartum participants [40, 65, 74, 77, 79] up to 2 years after birth [40]. Others researchers narrowed their review scope to samples diverse in ethnicities [75], or to those within the US [71]. One study also included HCPs [38], although the findings from HCPs are not reported in this review.

**Table 2:** Characteristics of included reviews. Note, the conclusion of whether a meta-analysis was performed was based on whether it was performed for the outcome of interest (i.e not for safety of vaccination). SS = sample size.

Review	Scope of review	Search dates	Number of included studies (and participants)	Inclusion criteria for participants	Barriers assessed (and number of studies cited)	Facilitators assessed (and number of studies cited)	Meta-analysis performed	JBI score
**Adeyanju et al, 2021 [64]**	Factors associated with seasonal Influenza vaccination	15 February 2020	11 (SS ranged from 198 to 11,752)	Pregnant	Risk perception (7) Safety of vaccine for mother and unborn child (5) Against vaccines in general (2) Conspiracy theory (1) Multiparity (1) Proximity to childbirth (1) Being of an ethnic minority (1) Low education (1) Being a refugee (1) Needle fears (1) Drug objections (1) Belief vaccine is not effective (3) Not knowing about the recommendation to be vaccinated (5) Hearing of bad experiences from social network (2)		No	2
**Azami et al., 2022 [80]**	Factors associated with COVID vaccination	October 2021	16 (total SS of 19, 219)	Pregnant		Month of study in 2021	Yes	18
**Badell et al., 2022 [78]**	Exploring COVID and COVID vaccination during pregnancy	November 2019 and March 2022	42 (SS ranged from 31 to 135 968	Pregnant	Younger age (3) Lower education/socioeconomic status (4) Lack of adherence to influenza vaccination recommendations (3) Being black or hispanic compared to being white (2) Concerns about side effects for baby (2) Worry vaccine rollout rushed for political reasons (1) Lack of safety and effectiveness data during pregnancy (1) Living in Russia, US, and Australia	Older age (10) Higher education (9) Urban living (3) Fertility treatment (3) Higher socioeconomic status (2) Being Asian (2) Previous influenza vaccine uptake (6) Higher level of trust in the healthcare system (3) Worry about COVID (5) Increased perceived risk of COVID (2) Knowledge of COVID (2) Reccomendation by HCP (4) Recieving counselling about vaccination from a HCP (1) Being a medical doctor (3) Belief in the importance of vaccines to their own country (1) Compliance with other COVID guidelines (3)	No	3
**Bisset et al., 2018 [66]**	Interventions to increase Pertussis and seasonal Influenza vaccination	04 August 2017	22 (no information on SS)	Pregnant		Midwives vaccinating (3) Information and education for patients (4)	No	3
**Callahan et al., 2021 [75]**	Interventions to increase Influenza vaccination	13 January 2021	13 (SS ranged from 1457 to 25,153)	Pregnant, at least 20% of participants identify as Black	Being black compared to other ethnicities (7) Worries of side effects (2) Low perceived risk (2) Harm to baby (1) Not recommended by a HCP (1) Dislike of vaccines (1) Negative previous vaccination experience (1)	Practice-based multicomponent intervention (2) Group prenatal care (1) Pamphlet with culturally diverse photographs (1) Text messaging (Text4baby) (1) Provider recommendation (7) Interpersonal support (1) History of Influenza immunisation (1) Greater belief in efficacy of the vaccine (1)	No	-4
**Ellingson et al., 2019 [76]**	Interventions to increase Influenza vaccination	2017 (no further date provided)	26 (no information on SS)	Pregnant		Interventions altering risk perceptions (19, but these were all part of combined interventions) Educational centred interventions (7) Higher perceived susceptibility (1) Perception of higher effectiveness (1) Providing transport to vaccination (1) Provider recommendation was the component of the package most strongly associated with vaccine receipt (1) Use of ’immunisation champions’ at the practice (2)	No	-3
**Galanis et al., 2022 [67]**	Factors associated with COVID vaccination	23 March 2022	11 (total SS of 70,3004)	Pregnant	Being Black or Hispanic (2) Mistrust in government (2) Diagnosis with COVID-19 during pregnancy (2) Worry about the safety and the side effects (2) Living in the most deprived areas (1)	Increased age (2) Trust in COVID-19 vaccines (2) Fear of COVID-19 during pregnancy (2) Having pregestational diabetes mellitus (2) Being White and Asian (2) Living in the least deprived areas (1)	Yes	5
**Januszek et al., 2021 [77]**	Factors associated with COVID vaccination	10 July 2021	9 (SS ranged from 17,871 and 152)	Pregnant and breastfeeding	Fear of harm to the foetus (6) Low socioeconomic status (1) Younger age Suspicion that the introduction of vaccines and advertising campaigns are politically motivated (3) Concerns about the safety (5) Concerns about side effects (including for the baby) (4)	Trust in the importance and effectiveness of the vaccine (3) Explicit communication about the safety of COVID vaccines during pregnancy (4) Acceptance of other vaccinations such as those for Influenza (2) Belief in the importance of vaccines/mass vaccination for one’s own country (1) Anxiety about COVID (2) Trust in public health agencies/health science (3) Compliance to mask guidelines (1) Older age (3) Higher education (5) Higher income/ employment (4)	No	-4
**Kilich et al., 2020 [40]**	Factors associated with Influenza, Pertussis and tetanus vaccination	22 November 2018	120 (total SS od 73,251)	Pregnant or have recently given birth (within 2 years)	Perceived as unsafe: pandemic (6) seasonal (7) Pandemic Influenza vaccination perceived to cause birth defects (2) Miscarriage (2) Side-effects (2) Knowledge of side effects (2) **Qualitative data:** Information gap/ lack of awareness (16) Concerns about narcolepsy, infertility, and autism (1) Unknown risks (6) Birth defects (3) Miscarriage (5) Concerns of side effects (11) Uncertainty about effectiveness (11) Being pregnant (3) Community rumours and cultural values (4) Preference for natural immunity/healthy lifestyle (5) Fear (13) Worry/anxiety (8), Responsibility for pregnancy outcomes (5) Uncertainty about risks (3) Fear of the unknown (4) Side effects (7) Pain (3) Fear of vaccine harms (12)	Accessibility and convenience (55) Personal values and lifestyle (43) Awareness of information regarding the specific vaccine or disease of focus (90) Social influences on vaccine use (109) Emotions related to vaccination (85) Risk perception (110) Perceptions of vaccine benefit 93) Personal vaccination history (80) HCP recommendation: Influenza (21), Pertussis (2) Information about the vaccine (4) Knowing there was a national vaccination policy in place (4) Prior vaccination history: seasonal (10) and pandemic Influenza (3), maternal pandemic Influenza vaccination history (2) Belief that pandemic Influenza benefits the mother (2) Seasonal vaccination perceived as effective (6) Believed to benefit the mother (6) Benefit to baby (7) Perceived susceptibility to seasonal Influenza (5) Belief it could be harmful to baby (3) **Qualitative data:** HCP recommendation (all qual studies) Offer of vaccination during antenatal visit (3) Being aware of maternal vaccination and/or the disease (17)	Yes	22
**Mohammed et al., 2019 [68]**	Interventions to increase Pertussis vaccination	01 January 2019	6 (SS ranged from 106 to 10,600)	Pregnant		iBook intervention (1) Combined interventions (1); education on Ipads, identifying a ’vaccine champion’, ensuring stock of vaccines.	No	19
**Nikpour et al., 2022 [69]**	Factors associated with COVID vaccination	11 July 2021	10 (total SS of 16, 696)	Pregnant		Being over 35 (3) High education level (3) High income level (2) High knowledge score of COVID infection (2)	Yes	-5
**Nindrea et al., 2022 [70]**	Factors associated with COVID vaccination	01 December 2021	12 (total SS of 14,444)	Pregnant	Low- and middle-income countries	Being over 35 years (4) Higher education (8) Sufficient information about the SARSCOV-2 vaccine (5) High perception of the vaccine (3) Good practice (2) Received Influenza vaccine last year (5) Being in the third trimester (3)	Yes	6
**Okoli et al., 2021 [65]**	Factors associated with Influenza vaccination	13 February 2020	34 (SS ranged from 100 to 1 862 705)	Pregnant and postpartum	Being Black in the US compared to being white (11) Smoking (8) Having cardiovascular disease	Older age (20) Being married (8) Nulliparity compared to multiparity (13) Previous receipt of Influenza vaccine (9) Living in a rural area (9) Being employed (4) Having prenatal care (3) Having chronic disease/s (6) Being asthmatic	Yes	11
**Parsons et al., 2021 [63]**	Interventions to increase Influenza vaccination	01 April 2020	10 (total SS of 9831)	Pregnant		Digital interventions (compared to non-digital or no intervention) (7) Any digital mode other than text-messaging (video, social media and iBook), compared to text messaging	Yes	19
**Rawal et al., 2022 [71]**	Factors associated with COVID vaccination	6 February, 2022	32 (11 examined factors associated with vaccination, SS ranged from 2002 to 135,968)	Pregnant, residing in the US	Concerns about side effects, sickness, allergy to the vaccine, and a perception that the vaccine is unnecessary (1) Refusal of the seasonal Influenza vaccine (2) Lack of provider counselling (1) Younger age (2) African American race (5) Hispanic ethnicity (4) Low education (1) Safety and effectiveness (3) Fears of birth defects (3) Unknown long-term health effects on children (3) Risk of pregnancy loss (3)	Study populations of higher income and education Receipt of the Influenza vaccine in the previous year (3) Communication with a medical professional about vaccines (3)	No	20
**Sarantaki et al., 2022 [79]**	Acceptance of and factors associated with COVID vaccination	September 2022	18 (SS ranged from 92 to 5307)	Pregnant or had recently given birth	Younger (2) Being Black or Hispanic (3)	Older age (2) Education level (7) Being with a partner (1) Occupational status (6): employed (4), full-time (2), and being a physician or HCP (2) Prescence of underlying diseases (2) Being in the third trimester (2) Previous reciept of influenza vaccination during pregnancy or within last year (6) Positive COVID-19 test (1) Higher level of knowledge around COVID-19 or the vaccine (2) Perception of vaccine as safe (2) and effective (2)	No	0
**Shamishirsaz et al., 2021 [73]**	Factors associated with COVID vaccination	22 May 2021	12 (total SS of 16,926)	Pregnant		Uptake of infleunza and/or TdaP during pregnancy	Yes	17
**Wilson et al., 2015 [38]**	Factors associated with Influenza, Pertussis and tetanus vaccination	22 April 2015	155 (SS ranged from 10 and 55,570)	Pregnant women, recently pregnant, and HCPs	Being of an Ethnic minority (27) Safety of vaccines in pregnancy (64) Concerns about the efficacy or the belief that the vaccine is not necessary (28) Low knowledge about the vaccines and/or the diseases they prevent (22) No recommendations from a HCP (17) Access/availability issues (6) Conflicting advice (3) Cost (2) Religion (1)	Agreement/advice from husband/partner (5) Recommendation from a HCW (most included studies)	No	-2
**Wong et al., 2016 [74]**	Interventions to increase Influenza vaccination	01 September 2014	11 (SS ranged from 126 to 21,292)	Pregnant and post-partum		Text message education and reminders (1) Education pamphlet (2), especially when combined with a verbal benefit statement (1) Education when part of a wider intervention e.g provider reminders, increasing access, standing orders (3)	No	18

## Findings

### Barriers and facilitators to COVID vaccination

#### Contextual influences

Black [67, 71, 78, 79] and Hispanic [78, 79] ethnicity and low socioeconomic status, either at the individual [77, 78] or area level [67], were reported as barriers to vaccination for COVID-19. There were mixed findings for the remaining sociodemographic variables [69, 73], particularly age [78–80] and not precited by review quality, although income and education were more frequently concluded to be positive predictors of vaccination [70, 71, 77–79]. Additional contextual predictors included suspicions around politically motivated vaccination campaigns [77, 78], possessing gestational diabetes [67] or fertility treatment [78], full-time employment [79], urban living [78] working as a HCP [78, 79], and being within the third trimester of pregnancy [70, 79].

#### Individual/ Social group influences

Concerns around the safety and side effects [67, 71, 77, 78], specifically for the unborn child [71, 77, 78], were the most frequently cited beliefs that were a barrier to vaccination, whilst previous receipt of other vaccinations was cited by the most reviews as a key facilitator [70, 71, 73, 77–79]. Other facilitators cited by more than one review were trust in the COVID-19 vaccine [67, 77] and healthcare system [78], knowledge of COVID-19 [69, 70, 78, 79], and compliance to other COVID-19 guidelines such as mask wearing and social distancing [70, 77, 78]. Less frequently cited barriers were concerns around effectiveness [71] and mistrust in the government [67], with less frequently cited facilitators including perception of the vaccine as safe and effective [79], diagnosis of COVID-19 during pregnancy [67, 79], fear of COVID-19 during pregnancy [67], and a sense of duty to one’s country [77, 78]. Whilst communication around the safety of the vaccine was reported as a facilitator by one review [77], knowledge was found to be a non-significant predictor by another [69].

#### Vaccine and vaccine-specific issues

Only one review [71] highlighted barriers around vaccination such as lack of a recommendation by a HCP, refusal of the Influenza vaccine, and allergies to vaccines as barriers. Another review [78] found recommendation by a HCP to be a key facilitator of vaccination, and receiving counselling about vaccination from a HCP to be an effective intervention.

### Barriers and facilitators to influenza and pertussis vaccination

#### Contextual influences

Black [65, 75] and ethnic minority ethnicities [38] were amongst the most commonly cited barriers of vaccination, with only one review finding no effect of ethnicity [65]. Evidence for education as a predictor of vaccination was mixed, with one review citing low education as a barrier [64], supported by one primary study, and another finding no association [65]. Older age, employment, and being married were found to predict vaccination, whilst no effect was found for income or socioeconomic status in general [65]. Concerning wider influences, awareness of a national policy to support vaccination resulted in a three-times higher probability of vaccination [40], with access and convenience another frequently cited facilitator [40]. One review each cited lack of convenience [38], and conspiracy theories [64] as barriers to vaccination.

Findings from the reviews of interventions found that when tailored to ethnic minorities, pamphlets were found to eliminate inequalities of vaccination by ethnicity [75]. Additionally, providing transport to the vaccination centre was reported to be an effective intervention [76], suggesting access and convenience as facilitators.

#### Individual/ Social group influences

Concerns about safety of the vaccination were the most commonly referred to barriers around individual/social group for Influenza and Pertussis [38, 40, 64, 75], both for the pregnant women [64], but mostly the unborn child [40, 64, 75]. Related barriers included emotions such as fear and worry [40]. Doubts about efficacy of the vaccine were also common [38, 64], and may be related to the influence of both low perceived risk of the illness [38, 64, 75] and perceived benefits of vaccination [38, 40]. Previous receiving of the vaccine was found to be a facilitator of maternal vaccination [40, 65, 75], increasing vaccination between three and five times [40], with an especially large effect on pandemic, and no effect on seasonal Influenza vaccination [40]. The influence of social groups could either act as a barrier [40, 64] or facilitator [38, 40] to vaccination. For example, hearing of bad experiences from one’s social network was cited as a barrier in a participants’ own decision to vaccinate [64], but encouragement from a partner acted as a facilitator [38]. The success of ‘immunisation champions’ reported by once review [75] support the findings of social encouragement and support as a facilitator.

Level of knowledge around the illness was cited as an important predictor of vaccination [38, 40]. However, the included intervention reviews found inconsistent evidence in support of education delivered by pamphlets [66, 74, 76], poster [66] or in-person sessions [66], and education provided through video did not significantly increase vaccination [66, 68, 75].

#### Vaccine and vaccine-specific issues

Recommendation from a HCP was the most frequently cited vaccine-specific facilitator of vaccination [38, 40, 75], increasing vaccination by 12 times for Influenza and 10 for Pertussis [40]. In support, the included intervention reviews found interventions involving a recommendation by a HCP [76], or increased contact (e.g. attending prenatal care) [65, 66, 75] with HCPs to increase vaccination uptake. Additional vaccine-specific barriers included a general aversion to vaccination [64, 75], a preference for natural immunity [40], pain [40], and needle fears [64].

#### Interacting factors

Two reviews discussed the influence of recommendation from a HCP for COVID-19, which was much more frequently cited for Pertussis and Influenza vaccination. Further, reviews investigating barriers and facilitators to COVID-19 were more likely to cite sociodemographic predictors and attitudes towards governing bodies, and those exploring Influenza and Pertussis were more likely to discuss social influences such as one’s partner and views of the community. Additionally, previous receiving of vaccination for other illnesses was a prominent predictor of COVID-19 vaccination and cited by a larger portion of included reviews than for Influenza and Pertussis. An awareness of the presence of the government in encouraging vaccinations was cited in relation to COVID-19 only. Only one review (rated the highest quality) assessed barriers and facilitators to seasonal and pandemic Influenza separately [40] and found that concerns around the safety of vaccination for the developing baby and side effects were particularly surrounding pandemic rather than seasonal Influenza.

Differences in COVID-19 vaccination rates by country [69, 70, 73, 77, 78] are possibly explicable by socioeconomic variables, as significantly lower rates were found in low- and middle-income countries [70], and as discussed, socioeconomic variables such as income and education were consistently cited in the included reviews. Country also interacted with effect of advice from a partner on maternal vaccination, namely through cultural gender norms and expectations [38].

Mode of delivery mattered for interventions to improve vaccination. Text messaging alone was consistently ineffective [72, 76], either to deliver education [75], to provide reminders [66], or both [74]. Video interventions were also found to be ineffective [68]. Generally, there was a pattern that in-person or physical rather than virtual interventions were more effective in increasing uptake, although one review focusing on the effect of digital interventions concluded that they were more effective than non-digital interventions [72]. Text-message reminders were effective when part of larger combined intervention that were practice-wide [74, 75], although it is difficult to identify their individual influence in increasing vaccination.

#### Findings from meta-analyses

Meta-analyses were only performed for Influenza and COVID-19 aside from one for Pertussis [40]. One review performed meta-analyses separately where possible [40]. As shown in Supplementary material 4 in S1 File, the findings for contextual influences of vaccination were mixed. Overall, Odd Ratios (ORs) for contextual factors were small in comparison to other predictors. The largest ORs observed were for education and age, although findings were inconsistent. Education was only explored in the context of COVID-19. The review reporting the lowest odds ratio (OR) was of high heterogeneity and low quality [69], and the remaining two found odds ratios of 1.33 [73] and 1.84 [70]. ORs for age were also highly inconsistent [65, 69, 70, 73, 80], ranging from 1.02 [80] to 2.01 [70], and not predicted by whether the vaccination was for Influenza or COVID-19, the quality of the included review, the quality of included studies, heterogeneity of included studies, or the possibility of publication bias. Employment [65, 73], being married [65, 73], income [69], being pregnant for the first time [73], having no children [65, 80], living in a rural area [65], and being Black ethnicity compared to other ethnicities in high income countries [65] had a minimal or no effect on vaccination.

Individual/social group influences generally predicted vaccination on a much larger magnitude, although again, the heterogeneity was large. The highest ORs were observed for recommendation from a HCP for seasonal Influenza and Pertussis [40]. Other predictors with a large magnitude included previous receipt of vaccination during pregnancy and in general and perceived benefit to the mother for pandemic Influenza [40], perceived benefit of vaccination and knowledge or information provision for seasonal Influenza [40], good practice (one’s general motivation to prevent illness and engagement in health behaviours to do so) for COVID-19 [70], and previous receipt of vaccination in general and Influenza [40, 65]. In terms of barriers to vaccination, perceiving the vaccine as unsafe and to cause birth defects or miscarriage, and knowledge of vaccine side effects predicted the lowest odds of vaccination uptake for Influenza [40]. Pre-existing comorbidities [73], history of infection [73], and being in the third trimester [70] exerted negligible effects on COVID-19 vaccination, comparable to smoking status [65] for Influenza.

The prediction of knowledge/information remained relatively similar across COVID-19 [69, 70] and pandemic Influenza [40], but was especially high for seasonal Influenza [40]. ORs for general previous receipt of vaccination were inconsistent [40, 65, 70, 80], with the lowest for COVID-19 [80] and the highest for pandemic Influenza [40]. A similar pattern was found for previous receipt of vaccination during pregnancy [40, 73], although the lowest OR was found for seasonal Influenza [40].

#### Overall acceptance rates

The only meta-analyses for vaccination acceptance rates were conducted for COVID-19 [67, 69, 73, 80], as shown in Supplementary Material 5 in S1 File. Perhaps predictably, actual vaccination rate was almost half the rate of acceptance of vaccination and intention to vaccine [67, 69], at 28% [73]. The reviews that did not apply meta-analysis found similar acceptance rates, ranging from 29.7% and 77.4% [77] and 3% and 65% [71] overall. One review found acceptance rates to be moderated by study quality, namely that as pooled estimates were higher for studies of moderate compared to low risk of bias, and for cross-sectional rather than cohort designs [67].

## Discussion

Nineteen reviews of maternal vaccination hesitancy and the effectiveness of interventions were retrieved. Knowledge and information, previous receipt, and concerns around the safety were found to be the most consistent predictors of vaccination during pregnancy. Demographic predictors such as ethnicity and age were frequently cited, although results could be inconsistent, and the magnitude of their effect was low. Mapping onto the model for determinants of vaccine hesitancy [21], individual and social group factors were found to be the largest and most consistent predictors, with only sparse evidence for vaccine and vaccine-specific issues.

There was consistent evidence that recommendation from a HCP predicted vaccination rates with a large magnitude, particularly for Influenza and Pertussis. This mirrors the strong consistent relationship found in the general population [81], which also applies to other preventative health behaviours such as screening. For example, discussion with a HCP has been cited as a key predictor of uptake of both breast [82, 83] and prostate [84] cancer screening, and endorsement from a HCP increased colorectal cancer screening by 6% [85]. Thus, interventions to improve attitudes of HCPs towards vaccination are essential in encouraging maternal vaccination. Of the included reviews that identified provider-focused interventions, providing training and education for HCPs [66, 68], reminding HCPs to discuss vaccination with patients [66, 74, 76], and a midwife delivery programme [66, 68] were found to be effective in increasing maternal vaccination. However, many strategies were part of combined interventions, therefore future interventions would benefit from testing their impact individually. Similarly, previous vaccination was a consistent predictor for vaccination of Influenza and COVID-19. This predictor aligns with the prominent prediction of past behaviour for vaccination [86] and a range of other health behaviours [87]. The influence of past behaviour is arguably indicative of habit, in which the behaviour becomes automatic. In support, habit and vaccination being thought of as an automatic process with little decision making involved significantly predict vaccination [88]. Relatedly, the current review found good practice of preventative healthcare to strongly predict maternal vaccination [70], which suggests that habits may translate to similar behaviours. Indeed, Fleig et al. found that the ability to transfer learning from one context into another mediated the relationship between habit strength and engaging in increased physical activity and improving diet together [89]. Therefore, future interventions should target those who have never been vaccinated before to ensure future vaccination adherence to other infectious diseases.

Whilst many barriers and facilitators remained constant across infectious diseases, there were some notable differences. For example, ethnicity and socioeconomic status were noted as predictors of COVID-19 vaccination, but inconsistently predicted Influenza and Pertussis vaccination. One explanation that the current review found is that mistrust in governing bodies is more relevant for COVID-19, which has been found to predict COVID-19 vaccination uptake [90]. Since mistrust in the government, scientists and medical professionals is higher in ethnic minorities [91, 92], the two factors may interact. However, mistrust was not found to fully mediate the relationship between ethnicity and COVID-19 vaccination [93]. Thus, investigation is required to explore the mediating factors for the increased influence of ethnicity for COVID-19 vaccination specifically. Additionally, predictors of Influenza vaccination varied between seasonal and pandemic strains. Whilst recommendation from a HCP, beliefs, knowledge and attitudes were more important for seasonal Influenza, previous vaccination was more important in predicting pandemic Influenza. Taken together with the finding that pandemic Influenza vaccination is predicted by previous receipt of seasonal Influenza vaccination [94], this supports the notion that there are numerous factors predicting vaccination for the first time, but after that, past behaviour becomes the strongest predictor of future vaccination, as discussed above [86]. However, the magnitude of the prediction of past behaviour did not translate from pandemic Influenza to COVID-19, indicating separate and distinct factors.

The inclusion of intervention reviews enriched the understanding of the current review of how barriers and facilitators may interact. For example, whilst most reviews reported knowledge as a key predictor of vaccination, the findings from the included effectiveness reviews indicate that education is rarely sufficient. Secondly, the included intervention reviews generally found person-to-person interventions to be more effective, potentially indicating social interaction as a facilitator for vaccination. Indeed, reviews of the general population have also found interventions delivered either in person or via telephone to be more effective than virtual or text messaging interventions [95, 96], demonstrating the influence of interpersonal support in encouraging vaccination. Finally, the current review found multi-component interventions to be most effective, reflecting the numerous barriers and facilitators identified and similar to findings from interventions to encourage vaccination in the general population [97]. Additionally, the current review suggests that when designing interventions for pregnant women, specific attention needs to be directed towards education and emotional regulation around the safety of the developing baby and subsequent infant.

This review has also provided valuable insights into the current state of the research into vaccine hesitancy during pregnancy. Firstly, the sociodemographic influences were most frequently discussed in the context of COVID-19. Whilst it may be that sociodemographic factors such as socioeconomic status are more relevant for COVID-19 vaccination, an alternate explanation is that the literature to explore its predictive factors is in its infancy. This may have created a bias towards investigating these factors as sociodemographic questions are relatively easy and quick to measure. Where barriers and facilitators outside of sociodemographic factors were explored, there lacked a quantitative analysis of findings. Related to this finding, recommendation from a HCP was minimally explored in the context of COVID-19. Interestingly, evidence relating to COVID-19 from primary studies is inconsistent [98, 99], with one finding that pregnant individuals resist recommendation from a HCP with a preference to delay vaccination until after pregnancy [99]. Therefore, more research is required to explore whether the prediction of recommendation from a HCP also applies to COVID-19 vaccination. Secondly, the current review found primary studies on COVID-19 to be judged as higher quality and with lower risk of bias than the literature on Influenza and Pertussis. A potential explanation is that none of the reviews on COVID-19 assessed risk of bias or quality using Cochrane tools or the GRADE framework, which is arguably the most rigorous assessment method as it considers numerous predictors of quality including risk of bias [100]. Alternatively, it may be that the literature on COVID-19 is of better quality because it is more recent, possibly due to an increased demand by funders for researchers to adhere to open science practices such as pre-registration [101]. Thirdly, the high ORs found for individual and social group factors in predicting Influenza vaccination were mainly driven by one review [40]. Although the review itself was judged to be of strong quality, publication bias was not assessed for most predictors, and the included primary studies were assessed to be of mixed quality. Therefore, conclusions for predictors based on only one review must be interpreted with caution.

There are several limitations to the current umbrella review that must be acknowledged. Most importantly, the degree of overlap of included primary studies was high especially for COVID-19 and intervention reviews, thus inflating the influence of repeated primary studies on the conclusions made. Nevertheless, a meta-analysis of meta-analyses was not performed, thus the problem of providing disproportionate statistical power to these studies was avoided [39]. Additionally, as the scope was to investigate barriers and facilitators on the level of pregnant individuals, this inherently missed some of the contextual and vaccine-specific determinants of vaccine hesitancy such as economics, health systems, and vaccine access [102]. Lastly, as the current review included multiple types of study and infectious disease, conclusions made for each domain are limited in their generalisability. However, this scope did allow for useful comparisons and contrasts to be made which can help inform the nuances of future targeted interventions.

## Conclusion

Although sociodemographic factors predict vaccine hesitancy, their influence is small compared to individual and social group influences. Policymakers should consider different levels of intervention to promote vaccination uptake and further research should directly compare predictive factors across COVID-19, and Influenza and Pertussis. In conclusion, more research, specifically quantitative synthesis, is needed to explore the barriers and facilitators to COVID-19 vaccination outside of sociodemographic variables, and the potential of interventions to improve COVID-19 vaccination rates. This will help inform on whether interventions to improve vaccination should be tailored depending on the infectious disease. Interventions to increase maternal vaccination during pregnancy and two years after childbirth should target first time vaccinators, be delivered through person-to-person contact, and be multi-component. Suggested components include targeting social norms and interpersonal support, knowledge, emotional regulation, and habit formation. Multi-level interventions should engage and provide education to HCPs, to encourage them to provide recommendations to pregnant women.

## Supporting information

S1 Checklist (DOCX)

S1 File (DOCX)
