# Supplementary material for: Barriers and facilitators of vaccine hesitancy for COVID-19, influenza, and pertussis during pregnancy and in mothers of infants under two years: An umbrella review
Source: PLoS One. 2023 Mar 2;18(3):e0282525. doi: 10.1371/journal.pone.0282525 (PMC9980804; doi:10.1371/journal.pone.0282525)
Supplement: S1 File — (DOCX) [file pone.0282525.s002.docx]

**Supplementary material**

**Supplementary material 1:** Specific search query applied for each database.

| **Database** | **Keywords** | **Search outcome** |
| --- | --- | --- |
| CINAHL (EBSCO) | ab(vaccin* OR immunis* OR immuniz* OR incoculat*)  AND  ab(Anxiet* OR doubt* OR trust* OR intent* OR dilemma* OR attitude* OR distrust OR mistrust OR controvers* OR objector* OR awareness OR dropout* OR Perception* OR misconception* OR uptake OR behavi*r OR exemption* OR refus* OR misinform* OR barrier* OR belief* OR fear* OR reject* OR oppos* OR choice* OR criticis* OR hesitanc* OR rumo*r OR delay OR accept* OR concern* OR knowledge OR confiden* OR decision OR anti-vaccin* OR predict* OR factors OR failure OR affect OR reason* OR utilis* OR utiliz* OR worry OR facilitate* OR enable* OR implement* OR frequency OR cause* OR willing* OR perspective* OR determine* OR react* OR indecision OR reluct*)  AND  ab(Influenza OR H1N1 OR H5N1 OR flu OR TIV OR IIV3 OR IIV4 OR COVID OR COVID19 OR "SARS-CoV-2" OR "SARS-CoV2" OR SARSCoV2 OR "SARSCoV-2" OR "SARS coronavirus 2" OR "2019 nCoV" OR "2019nCoV" OR "2019-novel CoV" OR "nCov 2019" OR "nCov 19" OR "severe acute respiratory syndrome coronavirus 2" OR "novel coronavirus disease" OR "novel corona virus disease" OR "corona virus disease 2019" OR "coronavirus disease 2019" OR "novel coronavirus pneumonia" OR "novel corona virus pneumonia" OR "severe acute respiratory syndrome coronavirus 2" OR ‘whooping cough’ OR ‘Pertussis’ OR ‘tdap’)  AND  ab(maternal OR antenatal OR prenatal OR pregnan* OR perinatal)  Filters: Publication date from 2009/01 to 2022/05; English Language | 2009-April 2022  n=769 |
| Health Research Collection and PsychArticles (ProQuest) | ab(vaccin* OR immunis* OR immuniz* OR incoculat*)  AND  ab(Anxiet* OR doubt* OR trust* OR intent* OR dilemma* OR attitude* OR distrust OR mistrust OR controvers* OR objector* OR awareness OR dropout* OR Perception* OR misconception* OR uptake OR behavi*r OR exemption* OR refus* OR misinform* OR barrier* OR belief* OR fear* OR reject* OR oppos* OR choice* OR criticis* OR hesitanc* OR rumo*r OR delay OR accept* OR concern* OR knowledge OR confiden* OR decision OR anti-vaccin* OR predict* OR factors OR failure OR affect OR reason* OR utilis* OR utiliz* OR worry OR facilitate* OR enable* OR implement* OR frequency OR cause* OR willing* OR perspective* OR determine* OR react* OR indecision OR reluct*)  AND  ab(Influenza OR H1N1 OR H5N1 OR flu OR TIV OR IIV3 OR IIV4 OR COVID OR COVID19 OR "SARS-CoV-2" OR "SARS-CoV2" OR SARSCoV2 OR "SARSCoV-2" OR "SARS coronavirus 2" OR "2019 nCoV" OR "2019nCoV" OR "2019-novel CoV" OR "nCov 2019" OR "nCov 19" OR "severe acute respiratory syndrome coronavirus 2" OR "novel coronavirus disease" OR "novel corona virus disease" OR "corona virus disease 2019" OR "coronavirus disease 2019" OR "novel coronavirus pneumonia" OR "novel corona virus pneumonia" OR "severe acute respiratory syndrome coronavirus 2" OR ‘whooping cough’ OR ‘Pertussis’ OR ‘tdap’)  AND  ab(maternal OR antenatal OR prenatal OR pregnan* OR perinatal)  Filters: Publication date from 2009/01/01 to 2022/22/05; English; Peer Reviewed | 2009-April 2022  n= 1964 |
| EPISTEMONIKOS | (title:(vaccin* OR immunis* OR immuniz* OR incoculat*)) OR (abstract:(vaccin* OR immunis* OR immuniz* OR incoculat*))  AND  (title:(Anxiet* OR doubt* OR trust* OR intent* OR dilemma* OR attitude* OR distrust OR mistrust OR controvers* OR objector* OR awareness OR dropout* OR Perception* OR misconception* OR uptake OR behavi*r OR exemption* OR refus* OR misinform* OR barrier* OR belief* OR fear* OR reject* OR oppos* OR choice* OR criticis* OR hesitanc* OR rumo*r OR delay OR accept* OR concern* OR knowledge OR confiden* OR decision OR anti-vaccin* OR predict* OR factors OR failure OR affect OR reason* OR utilis* OR utiliz* OR worry OR facilitate* OR enable* OR implement* OR frequency OR cause* OR willing* OR perspective* OR determine* OR react* OR indecision OR reluct*)) OR (abstract:(Anxiet* OR doubt* OR trust* OR intent* OR dilemma* OR attitude* OR distrust OR mistrust OR controvers* OR objector* OR awareness OR dropout* OR Perception* OR misconception* OR uptake OR behavi*r OR exemption* OR refus* OR misinform* OR barrier* OR belief* OR fear* OR reject* OR oppos* OR choice* OR criticis* OR hesitanc* OR rumo*r OR delay OR accept* OR concern* OR knowledge OR confiden* OR decision OR anti-vaccin* OR predict* OR factors OR failure OR affect OR reason* OR utilis* OR utiliz* OR worry OR facilitate* OR enable* OR implement* OR frequency OR cause* OR willing* OR perspective* OR determine* OR react* OR indecision OR reluct*))  AND  (title:(Influenza OR H1N1 OR H5N1 OR flu OR TIV OR IIV3 OR IIV4 OR COVID OR COVID19 OR "SARS-CoV-2" OR "SARS-CoV2" OR SARSCoV2 OR "SARSCoV-2" OR "SARS coronavirus 2" OR "2019 nCoV" OR "2019nCoV" OR "2019-novel CoV" OR "nCov 2019" OR "nCov 19" OR "severe acute respiratory syndrome coronavirus 2" OR "novel coronavirus disease" OR "novel corona virus disease" OR "corona virus disease 2019" OR "coronavirus disease 2019" OR "novel coronavirus pneumonia" OR "novel corona virus pneumonia" OR "severe acute respiratory syndrome coronavirus 2" OR ‘whooping cough’ OR ‘Pertussis’ OR ‘tdap’)) OR (abstract:(Influenza OR H1N1 OR H5N1 OR flu OR TIV OR IIV3 OR IIV4 OR COVID OR COVID19 OR "SARS-CoV-2" OR "SARS-CoV2" OR SARSCoV2 OR "SARSCoV-2" OR "SARS coronavirus 2" OR "2019 nCoV" OR "2019nCoV" OR "2019-novel CoV" OR "nCov 2019" OR "nCov 19" OR "severe acute respiratory syndrome coronavirus 2" OR "novel coronavirus disease" OR "novel corona virus disease" OR "corona virus disease 2019" OR "coronavirus disease 2019" OR "novel coronavirus pneumonia" OR "novel corona virus pneumonia" OR "severe acute respiratory syndrome coronavirus 2" OR ‘whooping cough’ OR ‘Pertussis’ OR ‘tdap’))  AND  (title:(maternal OR antenatal OR prenatal OR pregnan* OR perinatal)) OR abstract:( maternal OR antenatal OR prenatal OR pregnan* OR perinatal))  Filters: Publication date from 2009 to 2022; English | 2009-April 2022  n= 633 |

**Supplementary material 2:** Primary studies colour-coded using the following guide: high, very high or good quality or low/very low risk of bias = green, moderate or medium risk of bias/quality = orange, and weak quality or high or very high risk of bias = red. ‘NI’ = information could not be retrieved from the review.

**1a** (influenza and pertussis)

| **Review**  **Review JBI score**  **Quality/risk of bias assessment tool**  **Coding**  **Primary study** | **Adeyanju et al. (1)**  Low  GRADE framework  Strong= good | **Kilich et al. (2)**  Strong  JBI tools  Strong= good | **Okoli et al. (3)**  Strong  The National Institutes of Health (NIH) quality assessment tool  High= good |
| --- | --- | --- | --- |
| Abasi (2015) |  | Low |  |
| Adams (2020) |  |  | Good |
| Afridi (2005) |  |  |  |
| Agricola (2016) |  | Strong |  |
| Ahluwalia (2010) |  |  | Good |
| Ahluwalia (2014) |  |  | Good |
| Alessandrini (2019) |  |  | Good |
| Arnold (2019) |  |  | Good |
| Arriola (2016) |  |  | Good |
| Arriola (2018) |  | Strong |  |
| Ashfaq (2017) |  | Very low |  |
| Ball (2013) |  |  |  |
| Barrett (2018) |  | Strong | Good |
| Baroso Periera (2013) |  | Strong |  |
| Bartolo (2019) |  |  | Good |
| Beigi (2009) |  | Moderate |  |
| Ben Natan (2017) |  | Low |  |
| Bettinger (2016) |  | Strong |  |
| Bhaskar (2012) |  | Very low |  |
| Blanchard-Rohner (2012) | Moderate | Strong |  |
| Blondel (2012) |  | Moderate |  |
| Bodeker (2014) | Moderate | Strong |  |
| Bodeker (2015) | Low | Moderate |  |
| Buekens (1995) |  |  |  |
| Bonville (2015) |  |  |  |
| Campbell (2013) |  |  |  |
| Campbell (2015) |  | Moderate |  |
| Carlisle (2019) |  |  | High |
| Cassady (2012) |  | Strong |  |
| Castro-sanchez (2018) |  | Strong |  |
| Celikel (2014) |  | Low |  |
| Chalise (2019) |  |  | Good |
| Chamberlain (2015) |  | Strong |  |
| Chamberlain (2016) |  | Strong |  |
| Collins (2014) |  | Low |  |
| D’Alessandro (2018) |  | Strong |  |
| Deming (2002) |  |  |  |
| Dempsey (2016) |  | Moderate |  |
| Descamps (2019) | Moderate |  | Good |
| Dickin (1991) |  |  |  |
| Ding (2011) |  | Moderate |  |
| Ding (2019) |  |  | High |
| Ditsungeon (2016) |  | Strong |  |
| Dlugacz (2012) |  | Low |  |
| Donaldson (2015) |  | Low |  |
| Doraivelu (2019) |  |  | Good |
| Drees (2012) |  | Strong |  |
| Drees (2013) |  | Strong |  |
| Edmonds (2011) |  | Strong |  |
| Eppes (2013) |  | Low |  |
| Fabry (2011) |  | Strong |  |
| Fabry (2011)b |  |  |  |
| Fisher (2011) |  | Very low |  |
| Fleming (2018) |  | Very low |  |
| Freund (2011) |  |  |  |
| Fridman (2011) |  | Strong |  |
| Galazka (1995) |  |  |  |
| Ganle (2014) |  |  |  |
| Gaudelus (2016) |  | Low |  |
| Gauld (2016) |  | Moderate |  |
| Goldfarb (2011) |  | Very low |  |
| Gorman (2012) |  | Low |  |
| Gul (2016) |  | Very low |  |
| Ghaswalla (2019) |  |  | Good |
| Groom (2016) |  |  | Moderate |
| Gupta (1998) |  |  |  |
| Gyimah (2006) |  |  |  |
| Hallisay (2018) |  | Very low |  |
| Halperin (2014) |  | Strong |  |
| Hanquet (2011) |  |  |  |
| Hasnain (2007) |  | Moderate |  |
| Hassan (2016) |  | Very low |  |
| Hayles (2015) |  | Low |  |
| Healy (2015) |  | Moderate |  |
| Henninger (2013) |  | Strong |  |
| Henninger (2015) |  | Strong | Good |
| Hill (2018) |  | Very low |  |
| Honarvar (2012) |  | Moderate |  |
| Housey (2014) |  |  |  |
| Hu (2017) |  | Strong |  |
| Ishola (2013) |  |  |  |
| Jadoon (2017) |  | Very low |  |
| Jamil (1999) |  |  |  |
| Janagaraj (2019) |  |  | Good |
| Kang (2015) |  | Low |  |
| Kay (2012) |  | Moderate |  |
| Kfouri (2013) |  | Very low |  |
| Khan (2015) |  | Moderate |  |
| Kharbanda (2011) |  | Strong |  |
| Kissin (2011) |  |  |  |
| Kouassi (2012) |  | Strong |  |
| Koul (2014) |  | Very low |  |
| Krishnaswamy (2018) |  | Moderate |  |
| Kriss (2018) |  | Strong |  |
| Laenen (2015) |  |  |  |
| Larson Williams (2018) |  | Strong |  |
| Lau (2010) |  | Strong |  |
| Leddy (2009) |  |  |  |
| Legge (2014) |  |  | Good |
| Link-Gelles (2012) |  |  |  |
| Lohiniva (2014) |  | Strong |  |
| Lohm (2014) |  | Strong |  |
| Lotter (2018) |  | Strong | Good |
| Loubet (2016) |  | Strong | Moderate |
| Lu (2008) |  |  |  |
| Lynch (2012) |  | Strong |  |
| MacDougall (2016) |  | Moderate |  |
| Madico (1996) |  |  |  |
| Maertens (2018) |  |  | High |
| Maher (2013) |  | Strong | High |
| Maisa (2018) |  | Strong |  |
| Mak (2015) |  | Strong | High |
| Mak (2018) |  | Strong |  |
| Maltezou (2019) | Moderate |  | Good |
| Maral (2001) |  |  |  |
| Marsh (2014) |  | Strong |  |
| de Mattos (2003) |  | Very low |  |
| Maurici (2015) | Low |  |  |
| Maurici (2016) |  | Very low |  |
| Mayet (2017) |  | Strong |  |
| McCarthy (2012) |  | Low |  |
| McCarthy (2015) |  | Low |  |
| McQuaid (2016) |  | Strong |  |
| McQuaid (2018) |  | Moderate |  |
| Meharry (2013) |  | Strong |  |
| Mendoza-Sassi (2019) |  |  | High |
| Mersereau (2012) |  |  |  |
| Mitra and Manna (1997) |  | Very low |  |
| Mohammed (2018) |  | Strong | Good |
| Napolitano (2017) |  | Strong |  |
| Navaneetham (2002) |  |  |  |
| Offedu (2019) |  |  | High |
| O’Grady (2015) |  | Very low |  |
| Og Son (2014) |  | Very low |  |
| O’Shea (2018) | Low | Strong |  |
| Ozer (2010) |  | Moderate |  |
| Ozkaya Parlakay (2012) |  | Very low |  |
| Panda (2010) |  |  |  |
| Power (2009) |  |  |  |
| Prospero (2019) | Low |  |  |
| Puchalski (2014) |  | Moderate |  |
| Rahman (1982) |  |  |  |
| Regan (2016) |  | Strong |  |
| Richun (2018) |  | Strong |  |
| Roosihermiatie (2000) |  |  |  |
| Rowe (2019) |  |  | Good |
| Sakaguchi (2011) |  | Low |  |
| Schindler (2012) |  | Strong |  |
| Shono (2019) |  |  | High |
| Siddiqui (2017) |  | Moderate |  |
| Silverman and Grief (2001) |  | Very low |  |
| Song (2017) |  | Very low |  |
| Stark (2016) |  | Strong |  |
| Steelfisher (2011) |  | Moderate |  |
| Strassberg (2018) |  | Low |  |
| Taksdal (2013) |  | Strong |  |
| Tarrant (2013) |  | Strong |  |
| Tessier (2018) |  |  | Good |
| Thind (2005) |  |  |  |
| Tong (2008) |  | Strong |  |
| Tuells (2018) | Moderate | Strong |  |
| Ugezu (2018) |  | Low |  |
| Van Lier (2012) |  | Moderate |  |
| Varan (2014) |  | Strong |  |
| Vila-Candel (2016) | Moderate | Low |  |
| Vilca (2017) |  |  | Good |
| Vitek (2011) |  |  |  |
| Walker (2011) |  |  |  |
| White (2010) |  | Very low |  |
| Wilcox (2018) |  | Very low |  |
| Wilcox (2019) | Low | Moderate |  |
| Wiley (2013a) |  | Low |  |
| Wiley (2013b) |  | Low |  |
| Wiley (2015) |  | Strong |  |
| Wong (2015) |  |  |  |
| Ymba and Perrey (2003) |  | Very low |  |
| Yudin (2009) |  | Low |  |
| Yudin (2017) |  |  | Good |
| Yuet Sheung Yuen (2013) |  | Strong | Good |
| Yuen (2016) |  | Strong |  |
| Yuen and Xu |  | Very low |  |
| Zhao (2012) |  |  | Good |

**1b** (interventions for influenza and pertussis)

| **Review**  **Review JBI score**  **Quality/risk of bias assessment tool**  **Coding**  **Primary study** | **Bisset et al. (4)**  Low  Effective Public Health Practice Project’s (EPHPP) Quality Assessment Tool  Strong=good | **Mohammed et al. (5)**  Strong  JBI tools  (Applied same scoring as Kilich) strong=good | **Parsons et al. (6)**  Strong  Cochrane ROB tools  Low risk=good | **Wong et al. (7)**  Strong  Cochrane and GRADE or Newcastle-Ottawa  High=good |
| --- | --- | --- | --- | --- |
| Baxter (2013) | Weak |  |  |  |
| Bushar (2017) |  |  | Moderate |  |
| Chamberlain (2015) |  | Moderate | High |  |
| Chamberlain (2016) |  |  |  |  |
| Dexter (2012) | Weak |  |  |  |
| Frew (2014a) |  |  |  | Low |
| Frew (2014b) |  |  |  |  |
| Frew (2016) | Weak |  | High |  |
| Goodman (2015) | Strong |  | Low |  |
| Healey (2015) | Weak | Low |  |  |
| Hoppe (2011) |  |  |  |  |
| Jordan (2015) | Moderate |  | High |  |
| Jung (2016) |  |  |  |  |
| Klatt and Hopp (2012) | Moderate |  |  | Medium |
| Kris (2017) | Weak | Low |  |  |
| McCarthy (2012) | Moderate |  |  | Medium |
| McCarthy (2015) | Weak |  |  |  |
| Meharry (2014) | Moderate |  |  | Moderate |
| Mohammed (2018) |  | Moderate |  |  |
| Moniz (2013) | Strong |  | Low | High |
| Morgan (2015) | Weak | Low |  |  |
| Mouzoon (2010) | Weak |  |  | Medium |
| Ogburn (2007) | Moderate |  |  | Medium |
| O’Leary (2019) |  |  | High |  |
| Panda (2011) | Moderate |  |  | Low |
| Payakachat (2016) | Weak | Moderate |  |  |
| Pierson (2015) | Weak |  |  |  |
| Regan (2017) |  |  | Some concerns |  |
| Roussos-Ross (2020) |  |  |  |  |
| Sherman (2012) | Moderate |  |  | High |
| Stockwell (2014) | Moderate |  | Some concerns | Moderate |
| Wallis (2006) |  |  |  |  |
| Wong (2016) | Strong |  |  |  |
| Wootton (2018) |  |  |  |  |
| Yudin (2010) | Weak |  |  | Low |
| Yudin (2017) |  |  | High |  |

**1c** (COVID-19)

| **Review**  **Review JBI score**  **Quality/risk of bias assessment tool**  **Coding**  **Primary study** | **Azami et al. (8)**  Strong  Strong  Low=good | **Galanis et al. (9)**  Moderate  JBI tool  Low=good | **Nikpour et al. (10)**  Very low  Hoy tool  Low= good | **Nindrea et al. (11)**  Moderate  Newcastle-Ottowa scale  High=good | **Rawal et al. (12)**  Strong  JBI tools  Low=good | **Shamishiraz et al. (13)**  Strong  Newcastle-Ottowa scale  High= good |
| --- | --- | --- | --- | --- | --- | --- |
| Ahlers-Shcmidt (2020) |  |  |  |  | Moderate |  |
| Battarbee (2021) | Low |  |  | High |  | High |
| Battarbee (2022) |  |  |  |  | Moderate |  |
| Blakeway (2022) |  | Low |  |  |  |  |
| Carbone (2021) |  |  |  |  |  | High |
| Ceulemans (2021) | Low |  | Low |  |  | High |
| Desai (2021) |  |  |  |  | Moderate |  |
| Levy (2021) |  |  | Moderate | High |  | High |
| Gencer (2021) | Medium |  | Moderate |  |  |  |
| Geoghegan (2021) | Medium |  | Moderate | Medium |  | High |
| Goldshtein (2022) |  | Moderate |  |  |  |  |
| Goncu Ayhan (2021) | Medium |  |  | High |  | High |
| Hailemarium (2021) | Medium |  |  | High |  |  |
| Hirshberg (2021) |  |  |  |  | NI |  |
| Hoque (2020) | Medium |  |  |  |  | High |
| Hosokawa (2022) |  | Low |  |  |  |  |
| Huddleston (2021) |  |  |  |  | Low |  |
| Jayagobi (2021) | Medium |  |  |  |  |  |
| Levy (2021) | Medium |  |  |  | Moderate |  |
| Lipkind (2022) |  | Moderate |  |  |  |  |
| Mappa (2021) | Medium |  | Moderate |  |  | High |
| Mohan (2021) |  |  |  |  |  |  |
| Mose (2021) | Medium |  | Moderate | High |  |  |
| Nguyen (2021) | Low |  |  | High |  |  |
| Razzagi (2021) |  | Low |  |  | Moderate |  |
| Rottenstreich (2022) |  | Moderate |  |  |  |  |
| Siegel (2021) |  | Low |  |  |  |  |
| Skirrow (2021) | Medium |  | Moderate |  |  | High |
| Skjefte (2021) | Low |  | Low | High |  | High |
| Stock (2022) |  |  |  |  |  |  |
| Stuckelberger (2021) |  |  | Low | High |  |  |
| Sutton (2021) | Medium |  |  | High | NI | High |
| Sznajder (2022) |  |  |  |  | Moderate |  |
| Tao (2021) | Low |  | Low | High |  | High |
| Taubman (2022) |  | Moderate |  |  |  |  |
| Townsel (2021) |  |  |  |  | Low |  |
| Wainstock (2021) |  | Moderate |  |  |  |  |
| Wang (2021) |  |  |  | High |  |  |
| Wang (2022) |  |  |  |  | Moderate |  |
| UK Health security Agency (2021) |  | Low |  |  |  |  |

**Supplementary material 3**: display of the quality appraisal for question of the JBI critical appraisal tool, for each included review. Y = yes, N = no, U = unclear, and NA = not applicable.

| **Review** | Q1 | Q2 | Q3 | Q4 | Q5 | Q6 | Q7 | Q8 | Q9 | Q10 | Q11 | Overall |
| --- | --- | --- | --- | --- | --- | --- | --- | --- | --- | --- | --- | --- |
| Adeyanju et al., 2021 (1) | N | Y | N | Y | Y | N | N | Y | N | Y | Y | Low |
| Azami et al., 2022 (8) | Y | Y | Y | Y | Y | Y | Y | Y | Y | N |  | Strong |
| Badell et al., (2022) (14) | Y | Y | N | Y | N | N | N | Y | N | Y | Y | Very low |
| Bisset et al., 2018 (4) | N | Y | Y | Y | Y | N | N | Y | N | Y | U | Low |
| Callahan et al., 2021 (15) | N | U | U | Y | N | N | N | Y | N | Y | Y | Very low |
| Ellingson et al., 2019 (16) | N | N | Y | Y | U | N | U | U | N | Y | Y | Very low |
| Galanis et al., 2022 (9) | N | Y | N | Y | Y | U | U | Y | Y | Y | U | Moderate |
| Januszek et al., 2021 (17) | N | Y | N | N | N | NA | Y | Y | N | Y | N | Very low |
| Kilich et al., 2020 (2) | Y | Y | Y | Y | Y | Y | Y | Y | Y | Y | Y | Strong |
| Mohammed et al., 2019 (5) | Y | Y | Y | Y | Y | Y | U | Y | N | Y | Y | Strong |
| Nikpour et al., 2022 (10) | N | Y | N | N | Y | Y | Y | N | N | N | U | Very low |
| Nindrea et al., 2022 (11) | N | U | N | Y | Y | U | Y | Y | Y | Y | U | Moderate |
| Okoli et al., 2021 (3) | N | U | Y | Y | Y | Y | Y | N | Y | Y | Y | Strong |
| Parsons et al., 2021 (18) | Y | Y | Y | Y | Y | Y | U | N | Y | Y | Y | Strong |
| Rawal et al., 2022 (12) | Y | Y | Y | Y | Y | Y | Y | Y | N | Y | Y | Strong |
| Sarantaki et al., 2022 (19) | Y | Y | N | Y | N | N | N | Y | N | Y | N | Very low |
| Shamshirsaz et al., 2021 (13) | Y | Y | Y | Y | Y | U | Y | N | Y | Y | N | Strong |
| Wilson et al., 2015 (20) | U | U | Y | Y | N | N | U | U | N | Y | Y | Very low |
| Wong et al., 2016 (7) | N | U | Y | Y | Y | Y | U | Y | Y | Y | Y | Strong |

**Supplementary material 4: Table of meta-analyses**

| Category of predictor | Predictor | Vaccine type | Review | Heterogeneity (I squared, %) | CI (95%) | OR | JBI score |
| --- | --- | --- | --- | --- | --- | --- | --- |
| Contextual influences | Education | COVID-19 | Nikpour et al. (10) | 78.74 | 0.79-1.35 | 1.03 | -5 |
|  |  |  | Nindrea et al. (11) | 51.98 | 1.40–2.28 | 1.84 | 6 |
|  |  |  | Shamshirsaz et al. (13) | 92.5 | 0.48–3.71 | 1.33 | 17 |
|  | Employment status | COVID-19 | Shamshirsaz et al. (13) | 80.4 | 0.78–1,43 | 1.05 |  |
|  |  | Influenza | Okoli et al. (3) | 0.00 | 1.02-1.24 | 1.13 | 11 |
|  | Age | COVID-19 | Azami et al. (8) | 0.00 | 0.93-1.11 | 1.02 | 18 |
|  |  |  | Nikpour et al. (10) | 63.99 | 0.95-1.43 | 1.17 | -5 |
|  |  |  | Nindrea et al. (11) | 45.29 | 1.10–2.93 | 2.01 | 6 |
|  |  |  | Shamshirsaz et al. (13) | 59.4 | 0.88–1.26 | 1.05 | 17 |
|  |  | Influenza | Okoli et al. (3) | 96.4 | 1.07-1.20 | 1.13 | 11 |
|  | Being married | COVID-19 | Shamshirsaz et al. (13) | 93.00 | 0.66–2.44 | 1.27 | 17 |
|  |  | Influenza | Okoli et al. (3) | 0.00 | 1.07-1.15 | 1.11 | 11 |
|  | Income | COVID-19 | Nikpour et al. (10) | 72.30 | 0.80-1.75 | 1.18 | -5 |
|  | Primigravity | COVID-19 | Azami et al. (8) | 0.00 | 0.90-1.16 | 1.02 | 18 |
|  |  |  | Shamshirsaz et al. (13) | 75.4 | 0.95–1.36 | 1.14 | 17 |
|  | Being Black in comparison to other ethnicities | Influenza | Okoli et al. (3) | 68.1 | 0.71-0.83 | 0.77 | 11 |
|  | Living in a rural area | Influenza | Okoli et al. (3) | 56.3 | 1.05-1.14 | 1.09 |  |
|  | Nulliparity | Influenza | Okoli et al. (3) | 90.8 | 1.15-1.38 | 1.26 |  |
| Individual/social group influences | Knowledge/information | COVID-19 | Nikpour et al. (10) | 99.60 | 0.78- 8.34 | 2.55 | -5 |
|  |  | COVID-19 | Nindrea et al. (11) | 61.70 | 0.94–2.95 | 1.94 | 6 |
|  |  | Influenza (pandemic) | Kilich et al. (2) | 70.00 | 1.06-2.12 | 1.50 | 22 |
|  |  | Influenza (seasonal) | Kilich et al. (2) | 84.00 | 1.53–21.13 | 5.68 |  |
|  | Previous receipt of vaccination (in general) | COVID-19 | Azami et al. (8) | 0.00 | 0.71–1.06 | 0.87 | 18 |
|  |  |  | Nindrea et al. (11) | 74.92 | 2.09–3.35 | 2.72 | 6 |
|  |  | Influenza | Okoli et al. (3) | 96.3 | 3.14-7.57 | 4.88 | 11 |
|  |  | Influenza (pandemic) | Kilich et al. (2) | 88.00 | 2.44–12.37 | 5.49 | 22 |
|  |  | Influenza (seasonal) | Kilich et al. (2) | 63.00 | 2.49–5.73 | 3.78 |  |
|  | Previous receipt of vaccination (during a prev. pregnancy) | COVID-19 | Shamshirsaz et al. (13) | 82.7 | 1.37–6.73 | 3.03 | 17 |
|  |  | Influenza (pandemic) | Kilich et al. (2) | 83.00 | 1.99–41.76 | 9.12 | 22 |
|  |  | Influenza (seasonal) | Kilich et al. (2) | 76.00 | 0.71–3.24 | 1.51 |  |
|  | Pre-existing comorbidities | COVID-19 | Shamshirsaz et al. (13) | 0.00 | 0.84–1.13 | 0.97 | 17 |
|  |  | Influenza | Okoli et al. (3) | 85.8 | 1.17-1.44 | 1.30 | 11 |
|  | Perceived benefit of vaccine/ high perception (general) | COVID-19 | Nindrea et al. (11) | 0.00 | 2.13–3.27 | 2.70 | 6 |
|  |  | Influenza (pandemic) | Kilich et al. (2) | 0.00 | 0.69-1.51 | 1.02 | 22 |
|  |  | Influenza (seasonal) | Kilich et al. (2) | 80.00 | 3.49-14.93 | 7.22 |  |
|  | Perceived benefit to baby | Influenza (pandemic) | Kilich et al. (2) | 98.00 | 0.96-21.44 | 4.53 |  |
|  |  | Influenza (seasonal) | Kilich et al. (2) | 44.00 | 1.18-2.57 | 1.74 |  |
|  | Perceived benefit to mother | Influenza (pandemic) | Kilich et al. (2) | 0.00 | 2.90– 24.61 | 8.44 |  |
|  |  | Influenza (seasonal) | Kilich et al. (2) | 82.00 | 2.19–5.51 | 3.47 |  |
|  | Perceived susceptibility to illness | Influenza (pandemic) | Kilich et al. (2) | 95.00 | 0.56-2.19 | 1.11 |  |
|  |  | Influenza (seasonal) | Kilich et al. (2) | 35.00 | 1.26– 2.47 | 1.76 |  |
|  | Recommendation from a HCP | Influenza (pandemic) | Kilich et al. (2) | 92.00 | 3.12-14.64 | 6.76 |  |
|  |  | Influenza (seasonal) | Kilich et al. (2) | 92.00 | 6.80–21.23 | 12.02 |  |
|  |  | Pertussis | Kilich et al. (2) | 0.00 | 5.49–19.43 | 10.33 |  |
|  | Vaccine perceived as unsafe | Influenza (pandemic) | Kilich et al. (2) | 89.00 | 0.09–0.29 | 0.16 |  |
|  |  | Influenza (seaonsal) | Kilich et al. (2) | 84.00 | 0.11–0.44 | 0.22 |  |
|  | Vaccine perceived to cause side effects | Influenza (pandemic) | Kilich et al. (2) | 0.00 | 0.23– 0.81 | 0.44 |  |
|  |  | Influenza (seasonal) | Kilich et al. (2) | 96.00 | 0.27-1.16 | 0.55 |  |
|  | Vaccine perceived to cause birth defects | Influenza (pandemic) | Kilich et al. (2) | 14.00 | 0.09–0.40 | 0.19 |  |
|  | Vaccine perceived to cause miscarriage | Influenza (pandemic) | Kilich et al. (2) | 64.00 | 0.10–0.38 | 0.19 |  |
|  | Knowledge of vaccine side effects | Influenza (pandemic) | Kilich et al. (2) | 0.00 | 0.21–0.34 | 0.27 |  |
|  | Belief illness could harm baby | Influenza (seasonal) | Kilich et al. (2) | 78.00 | 1.37–9.94 | 3.70 |  |
|  | Receiving prenatal care | Influenza | Okoli et al. (3) | 98.00 | 2.25-5.02 | 3.36 | 8 |
|  | Smoking status | Influenza | Okoli et al. (3) | 86.3 | 0.66-0.96 | 0.80 | 8 |
|  | Good practice | COVID-19 | Nindrea et al. (11) | 0.00 | 7.42–10.56 | 8.99 | 6 |
|  | Being in the third trimester | COVID-19 | Nindrea et al. (11) | 0.00 | 1.10–1.60 | 1.35 | 6 |
|  | History of infection | COVID-19 | Shamshirsaz et al. (13) | 78.00 | 0.49–1.42 | 0.83 | 8 |

**Supplementary material 5: Table of meta-analyses of acceptance rates for COVID-19**

| Review | Outcome (as described) | Number of included studies | Heterogeneity (I squared %) | 95% Confidence Interval (%) | Pooled proportion of acceptance rates (%) |
| --- | --- | --- | --- | --- | --- |
| Azami et al. (8) | Acceptance | 16 | 98.19 | 47.64–59.24 | 53.46 |
| Galanis et al. (9) | Vaccination rate | 11 | 99.98 | 19-37 | 28 |
| Nikpour et al. (10) | Acceptance | 10 | 99.05 | 45-62 | 54 |
| Sarantaki et al. (19) | Acceptance | 18 | 99.29 | 0.44–0.61 | 53 |
| Shamshirsaz et al. (13) | Intention | 12 | 99.28 | 38 - 57 | 47 |

**References**

1. Adeyanju G, E E, L K, T R, IBM S, MG H, et al. Determinants of influenza vaccine hesitancy among pregnant women in Europe: a systematic review. European journal of medical research. 2021;26(1):116.

2. Kilich E, Dada S, Francis MR, Tazare J, Chico RM, Paterson P, et al. Factors that influence vaccination decision-making among pregnant women: a systematic review and meta-analysis. PloS one. 2020;15(7):e0234827.

3. Okoli GN, Reddy, V. K., Al‐Yousif, Y., Neilson, C. J., Mahmud, S. M., & Abou‐Setta, A. M. Sociodemographic and health-related determinants of seasonal influenza vaccination in pregnancy: A systematic review and meta-analysis of the evidence since 2000. Acta obstetricia et gynecologica Scandinavica. 2021;100(6):997-1009.

4. Bisset KA, Paterson P. Strategies for increasing uptake of vaccination in pregnancy in high-income countries: A systematic review. Vaccine. 2018;36(20):2751-9.

5. Mohammed H, McMillan M, Roberts CT, Marshall HS. A systematic review of interventions to improve uptake of pertussis vaccination in pregnancy. PloS one. 2019;14(3):e0214538.

6. Parsons J, Griffiths SE, Thomas N, Atherton H. How effective are digital interventions in increasing flu vaccination among pregnant women? A systematic review and meta-analysis. J Public Health. 2021;10.

7. Wong VW, Lok KY, Tarrant M. Interventions to increase the uptake of seasonal influenza vaccination among pregnant women: A systematic review. Vaccine. 2016;34(1):20-32.

8. Azami M, Nasirkandy MP, Esmaeili Gouvarchin Ghaleh H, Ranjbar R. COVID-19 vaccine acceptance among pregnant women worldwide: A systematic review and meta-analysis. PloS one. 2022;17(9):e0272273.

9. Galanis P, Vraka I, Siskou O, Konstantakopoulou O, Katsiroumpa A, Kaitelidou D. Uptake of COVID-19 vaccines among pregnant women: a systematic review and meta-analysis. medRxiv. 2022:2022.04. 01.22273296.

10. Nikpour M, Sepidarkish M, Omidvar S, Firouzbakht M. Global prevalence of acceptance of COVID-19 vaccines and associated factors in pregnant women: a systematic review and meta-analysis. Expert Review of Vaccines. 2022(just-accepted).

11. Nindrea RD, Djanas D, Darma IY, Hendriyani H, Sari NP. The risk factors and pregnant women's willingness toward the SARS-CoV-2 vaccination in various countries: A systematic review and meta-analysis. Clinical Epidemiology and Global Health. 2022:100982.

12. S R, RL T, RH S, HN Y. COVID-19 Vaccination among Pregnant People in the U.S.: A Systematic Review. American journal of obstetrics & gynecology MFM. 2022:100616.

13. Shamshirsaz AA, Hessami, K., Morain, S., Afshar, Y., Nassr, A. A., Arian, S. E., ... & Aagaard, K. Intention to receive COVID-19 vaccine during pregnancy: A systematic review and meta-analysis. American journal of perinatology. 2021.

14. Badell ML, Dude CM, Rasmussen SA, Jamieson DJ. Covid-19 vaccination in pregnancy. bmj. 2022;378.

15. Callahan AG, Coleman-Cowger, V. H., Schulkin, J., & Power, M. L. . Racial disparities in influenza immunization during pregnancy in the United States: A narrative review of the evidence for disparities and potential interventions. Vaccine. 2021.

16. Ellingson MK, Dudley, M. Z., Limaye, R. J., Salmon, D. A., O’Leary, S. T., & Omer, S. B. Enhancing uptake of influenza maternal vaccine. Expert review of vaccines. 2019;18(2):191-204.

17. Januszek SM, Faryniak-Zuzak, A., Barnaś, E., Łoziński, T., Góra, T., Siwiec, N., ... & Kluz, T. The approach of pregnant women to vaccination based on a COVID-19 systematic review. Medicina. 2021;57(9):977.

18. Parsons J, Atherton H. How effective are digital interventions in increasing flu vaccination amongst pregnant women? A systematic review protocol. Systematic reviews. 2020;9(1):1-6.

19. Sarantaki A, Kalogeropoulou VE, Taskou C, Nanou C, Lykeridou A. COVID-19 Vaccination and Related Determinants of Hesitancy among Pregnant Women: A Systematic Review and Meta-Analysis. Vaccines. 2022;10(12):2055.

20. Wilson RJ, Paterson P, Jarrett C, Larson HJ. Understanding factors influencing vaccination acceptance during pregnancy globally: a literature review. Vaccine. 2015;33(47):6420-9.
